# Inference of Indium Competition on the Optical Characteristics of GaAs/In*_x_*Ga_1−*x*_As Core–Shell Nanowires with Reverse Type-I Band Alignment

**DOI:** 10.3390/ma18174030

**Published:** 2025-08-28

**Authors:** Puning Wang, Huan Liu, Yubin Kang, Jilong Tang, Qun Hao, Zhipeng Wei

**Affiliations:** 1 State Key Laboratory of High-Power Semiconductor Laser, School of Physics, Changchun University of Science and Technology, Changchun 130022, China; puning_wang@163.com (P.W.); yubinkangcust@126.com (Y.K.); jl_tangcust@163.com (J.T.); qhao@bit.edu.cn (Q.H.); 2 Department of Electrical and Electronic Engineering, Southern University of Science and Technology, Shenzhen 518055, China; 12149014@mail.sustech.edu.cn

**Keywords:** III-V semiconductors, core–shell nanowires, composition graded layer, optical property

## Abstract

One-dimensional GaAs/InGaAs core–shell nanowires (NWs) with reverse type-I band alignment are promising candidates for next-generation optoelectronic devices. However, the influence of composition gradients and atomic interdiffusion at the core–shell interface on their photoluminescence (PL) behavior remains to be clarified. In this work, GaAs/In*_x_*Ga_1−*x*_As NW arrays with different indium (In) compositions were prepared using molecular beam epitaxy (MBE), and their band alignment and optical responses were systematically investigated through power and temperature-dependent PL spectra. The experiments reveal that variations in the In concentration gradient modify the characteristics of potential wells within the composition graded layer (CGL), as reflected by distinct PL emission features and thermal activation energies. At elevated temperatures, carrier escape from these wells is closely related to the observed PL saturation and emission quenching. These results provide experimental insight into the relationship between composition gradients, carrier dynamics, and emission properties in GaAs/InGaAs core–shell NWs, making them promising candidates for high-performance nanoscale optoelectronic device design.

## 1. Introduction

Semiconductor nanowires (NWs) have attracted significant attention due to their unique one-dimensional (1D) structure, enabling a wide range of applications in electronics, optoelectronics, and energy conversion devices [1,2,3]. Among III-V semiconductor NWs, ternary InGaAs NWs stand out as promising candidates for fiber-optic communications, owing to their tunable composition and, consequently, adjustable bandgap covering the crucial 1.3–1.55 μm telecommunication window. However, the direct growth of high-indium-content InGaAs NWs remains challenging, including compositional or morphological inhomogeneities [4]. InGaAs NWs can be grown via the vapor–liquid–solid (VLS) mechanism, in which a seed particle acts as a catalytic site to collect growth species during metal–organic chemical vapor deposition (MOCVD) or molecular beam epitaxy (MBE) [5,6,7]. When InGaAs NWs with high In composition are grown directly on the GaAs or Si substrate, the huge mismatch stress will lead to serious dislocations, defects, and even three-dimensional island or NW tapering growth [8,9]. In the early research conducted by Kim et al., although In-rich InGaAs NWs were grown on GaAs (111) B substrates using a Au-catalyzed VLS approach via MOCVD, the surface diffusion, solubility, and incorporation kinetics of dividual group-III element still need to be solved [10]. Furthermore, Joyce et al. and Shin et al. lowered both the growth temperature and the V/III ratio during MOCVD to suppress NW tapering and compositional inhomogeneity to some extent [11,12]. As for Au-free self-catalyzed MBE growth of InGaAs NWs, the group-III (Ga, In) droplet time delay may complicate the formation of sharp composition-tuned NW heterostructures during the growth process [13]. Also, the incorporation of In was limited to a significantly low composition (5%) in the bulk of InGaAs NWs and formed an In-rich shell structure, even though it improved the growth conditions [14].

To address these limitations, radial core–shell NWs architectures, particularly those featuring reverse type-I band alignment in GaAs/InGaAs structures, have emerged as a promising solution [15,16]. In this configuration, the GaAs core serves as a robust growth template and mechanical support [17]. Crucially, the inherent high surface-to-volume ratio and radial flexibility of NWs empower them to accommodate significantly greater lattice mismatch than bulk materials or thin films, effectively suppressing the formation of catastrophic defects [18]. These positive properties enable the MBE growth of GaAs/InGaAs core–shell NWs with exceptional crystal quality, even allow incorporating higher In compositions in the shell, which are difficult to realize on homogeneous InGaAs NWs of comparable In content [19]. Beyond preserving the tunable bandgap characteristic inherent to the GaAs/InGaAs material system, the reverse type-I band alignment plays a pivotal role in carrier management [20]. In this alignment, both electrons and holes are energetically driven towards the lower bandgap material [21]. Consequently, carriers are spatially confined within the outer InGaAs shell layer. This localization of charge carriers near the NW surface offers a substantial advantage for surface-sensitive applications [22]. Carriers can readily participate in surface chemical reactions or interact with adsorbates and the surrounding environment. As a result, processes such as photocatalysis and sensing can achieve significantly enhanced efficiency [23,24]. This makes the reverse type-I band alignment GaAs/InGaAs core–shell NWs particularly well-suited for advanced optoelectronic devices demanding efficient carrier extraction or surface interactions, including high-performance photodetectors, photocarrier collection systems, and novel catalytic [25,26,27,28]. Furthermore, due to the large lattice mismatch between GaAs core and high composition InGaAs shell, such core–shell NWs bring the chances to realize largely different compositions or bandgaps between core and shell and even alloy NWs with composition graded layer (CGL) on a same substrate. Therefore, high indium composition GaAs/InGaAs core–shell NWs with CGL offer significant potential for new material platform used in super broadly tunable nano-lasers, color engineered display and lighting, multispectral detectors, and full spectrum solar cells [29,30].

In this manuscript, aligned GaAs/InGaAs core–shell NWs arrays with In composition of 0.3 and 0.5 were successfully grown via a self-catalyzed VLS mechanism within an ultrahigh vacuum MBE. The morphological properties of GaAs/InGaAs core–shell NWs were systematically investigated. According to photoluminescence (PL) measurements, a peak is found in which photon energy is between the core and shell. Furthermore, power and temperature-dependent optical measurements were performed to analyze the bandgap and the behavior of photogenerated carriers, which proved that the peak is related to CGL. Spectroscopic analysis enables clear identification of band alignment and carrier dynamics among the core, shell, and the CGL in the GaAs/InGaAs reverse type-I band alignment core–shell NWs.

## 2. Materials and Methods

### 2.1. Growth of Nanowires

The GaAs/InGaAs core–shell NWs were grown on Si (111) substrate by solid source MBE system (DCA P600, DCA Instruments Oy, Turku, Finland). Specific growth parameters can be referred to in our previous publication [31]. For simplicity, GaAs/In_0.3_Ga_0.7_As core–shell NWs and GaAs/In_0.5_Ga_0.5_As core–shell NWs are indexed as Sample A and Sample B, respectively. The grown conditions of GaAs core of two samples are the same. The In component in the InGaAs shell can be well controlled by adjusting the flux ratio of the Ga and In flux. For sample A, the beam equivalent pressure (BEP) of Ga was set to 6.2 × 10^−8^ Torr and the In was set to 2.7 × 10^−8^ Torr. For sample B, the BEP of Ga maintained at 6.2 × 10^−8^ Torr, and the In increased to 5.4 × 10^−8^ Torr. The growth temperature was fixed at 520 °C for two samples. During the material growth, the other growth conditions are the same as the previous report [31].

### 2.2. Characterizations of Morphological and Optical Properties

The morphologies of the samples were characterized using scanning electron microscopy (SEM, ZEISS Gemini 300 system, ZEISS, München, Germany). Further structural and crystallinity analyses were performed using high-angle annular dark field scanning transmission electron microscopy (HAADF-STEM) and high-resolution transmission electron microscopy (HRTEM, FEI Tecnai G2-F20 microscope operated at 200 kV, FEI Company, Hillsboro, OR, USA) equipped with energy dispersive X-ray spectroscopy (EDS) detectors. A continuous-wave semiconductor diode laser operating at 660 nm served as the excitation source for all PL measurements. For low temperature power-dependent PL measurements, the samples were placed inside a closed-cycle helium cryostat with quartz windows with automatic temperature control. The excitation power was set to 30 mW for temperature-dependent PL measurements, while for power-dependent PL measurements the temperature was fixed at 50 K. The optical signal was dispersed by a monochromator (Andor Shamrock 303i, Andor Technology Ltd., Belfast, Northern Ireland, UK) and detected by a charge-coupled device (CCD, iDus DU490A) camera, with a spectral sensitivity range of 500 to 1700 nm.

## 3. Results and Discussion

**Figure 1:**
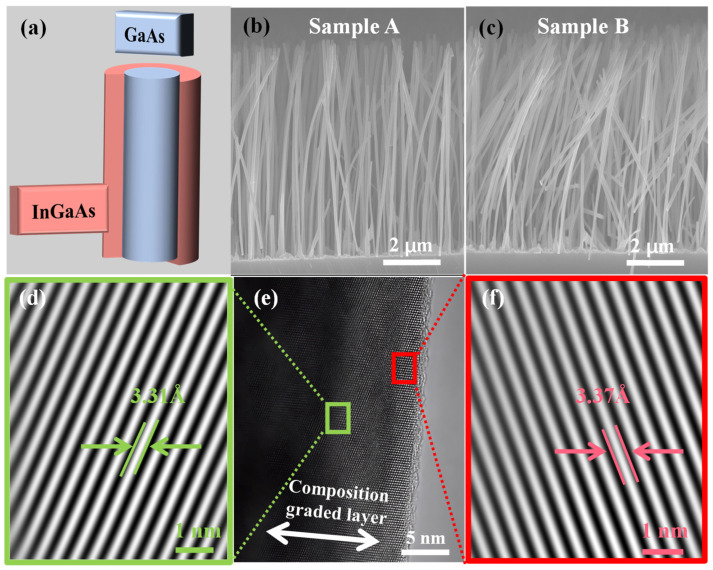
(**a**) The schematic structure of a NW of two samples. (**b**) Side-view SEM images of Sample A and **(c)** Sample B. (**d**) IFFT image of CGL of (**e**). (**e**) HRTEM image of Sample B. (**f**) IFFT image of In0.5Ga0.5As shell of (**e**).

The schematic structure of a GaAs/InGaAs core–shell NW is shown in Figure 1a, and both Sample A and B are of this structure. The SEM images of Sample A and B are illustrated in Figure 1b and Figure 1c, respectively. SEM characterization reveals the morphological properties of two samples are well-aligned on the Si substrate and exhibit excellent uniformity. The average diameter and length of both samples are 138.3 ± 2.0 nm and 7.9 ± 0.3 μm, respectively. The GaAs core measures approximately 80 nm in diameter, while the InGaAs shell has a thickness of about 30 nm. Notably, Sample B exhibits pronounced NW bending compared to Sample A. This bending can be attributed to residual strain and local compositional variations caused by the higher In content in the shell layer of Sample B [32]. Our previous work has reported the core–shell structure, In composition, and the presence of strain between the core and shell in Sample A [31]. To compare the lattice structure of Samples A and B, a HRTEM image of Sample B is presented in Figure 1e. Figure 1d and Figure 1f show the inverse fast Fourier transform (IFFT) images of the green and red rectangular regions highlighted in Sample B, representing areas slightly farther from, and closer to, the shell, respectively. The interplanar spacings (*d*) in the green and red regions are approximately 3.31 Å and 3.37 Å, respectively, along the same crystallographic plane. According to Bragg’s law, *λ* = 2*d* sin*θ*

(1) λ=2d sinθ

in which the wavelength (*λ*) is 1.5418 Å [33]. The 2*θ* values for the (111) reflections are 27.3° for GaAs (JCPDS No. 32-0389) and 25.4° for InAs (JCPDS No. 15-0869), as identified by the Joint Committee on Powder Diffraction Standards (JCPDS) database. The theoretical interplanar spacing (*d*) for In_0.5_Ga_0.5_As is 3.382 Å. The relationship between the lattice constant (*a*) and *d* is defined as follows [34]:

(2) $$a=d_{hlk}\sqrt{h^{2}+k^{2}+l^{2}}$$

where *h*, *k*, and *l* are the Miller indices. And the lattice constants of In*_x_*Ga_1−*x*_As as a function of x is followed by a linear relationship which described by Vegard’s law [35]:

(3) $$a_{{{In}_{x}Ga}_{1-x}As}=\left(1-x\right)a_{GaAs}+xa_{InAs}$$

where *x* is 0.5 in Sample B. The theoretical lattice constant of In_0.5_Ga_0.5_As is 5.858 Å. Experimentally, the lattice constants measured in two different regions of the shell in Sample B are 5.837 Å and 5.733 Å, respectively. Comparison of the theoretical and experimental values indicates the presence of compressive strain in the In_0.5_Ga_0.5_As shell. Moreover, the variation in lattice constants between these two regions suggests the existence of an indium inhomogeneity region or a CGL.

**Figure 2:**
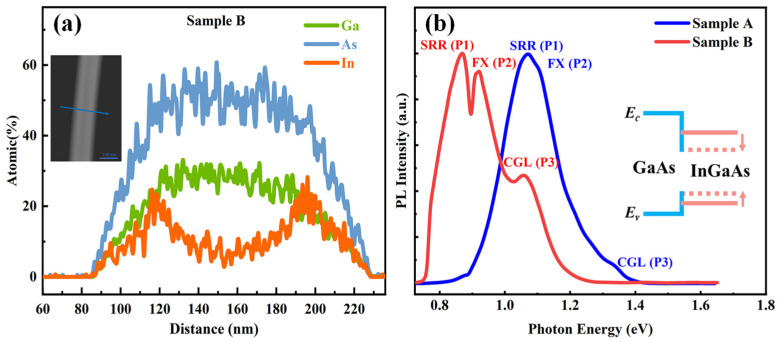
(**a**) EDX spectrum of the cross-section from sample B. The inset shows cross-sectional HAADF image of the Sample B with EDS line scan. (**b**) Normalized low-temperature (50 K) PL spectra of Sample A and Sample B. The inset shows the schematic image of core–shell band alignments of GaAs and InGaAs with different In composition.

To determine the elemental distribution of Sample B, the EDS spectra is presented in Figure 2a. The inset displays a cross-sectional HAADF image of Sample B with an EDS line scan. The results indicate a slight inhomogeneity in the In distribution, with an average indium atomic percentage of approximately 50% in Sample B [36]. Normalized low temperature (50K) PL measurements in two samples under 30 mW excitation are shown in Figure 2b, and the inset is the related schematic image of band structure. The discontinuity near 0.89 eV is attributed to a water absorption band at 1.4 μm [37]. The intensity drop near 0.78 eV results from the cutoff wavelength of the CCD. Both samples exhibit three peaks, labeled P1, P2, and P3 in order of increasing photon energy. All three peaks are located at energies lower than that of GaAs NWs (1.519 eV), suggesting that carrier recombination mainly occurs within regions of narrower bandgap, rather than in the GaAs core. This behavior is indicative of a reverse type-I band alignment. P1 and P2 originate from the surface related recombination (SRR) and free exciton (FX) emission, respectively, which have identical origins in Sample A and B [31]. P3 appears at approximately 1.33 and 1.06 eV in Samples A and B, respectively. These peaks are likely associated with emission from the CGL, as their energies fall between those of the GaAs core and the InGaAs shell in the respective samples.

**Figure 3:**
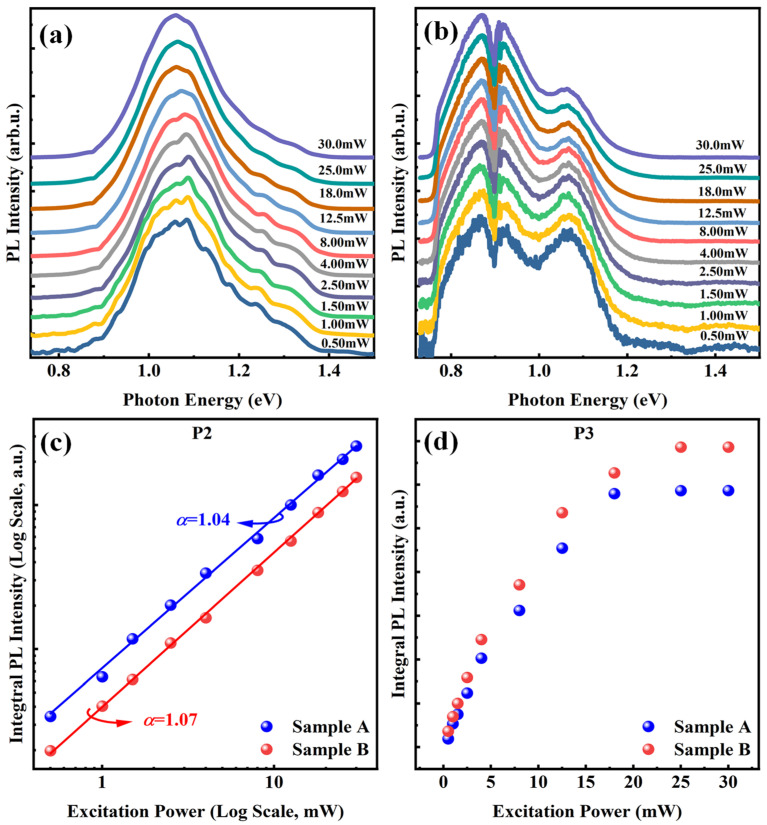
(**a**) Evolution of the PL spectra measured at 50 K under different power excitation of Sample A and (**b**) Sample B. (**c**) Power dependents of the integral PL intensity of P2 and (**d**) P3 of Sample A and B.

To further investigate the optical properties of two samples, normalized power-dependent PL spectra acquired at 50 K for both samples were compared, as shown in Figure 3a,b. The excitation power spanned from 0.5 to 30 mW. A systematic discussion of P1 was provided in our previous study; so, here we mainly focus on the variations in peaks P2 and P3 in both samples [31]. As observed, peaks P1 and P2 are dominant at all excitation powers in Sample A, while P3 is not a main peak and can only be distinguished at low excitation power. In contrast, all three peaks are clearly observed in Sample B. At the lowest excitation power, P3 exhibits a full width at half maximum (FWHM) and an intensity comparable to those of the other peaks. Normally, if the sample has a uniform composition distribution, carriers tend to recombine at the lowest energy band levels, rather than at intermediate levels. However, in our case, as the excitation power increases, the intensities of P1 and P2 increase more rapidly than that of P3 and become dominant in Sample B. This competitive behavior in PL intensity is likely caused by the formation of a wide potential well and barrier within the CGL, which influences carrier dynamics. At low excitation power (~0.5 mW), the density of photo-generated carriers is low, so most carriers are captured by the potential well formed in the CGL, resulting in comparable PL intensities for all three peaks. As the excitation power increases, the number of photo-generated carriers rises rapidly, and most of them recombine as free carriers. Consequently, the intensity of P2 increases rapidly and gradually becomes dominant, while the relative intensity of P3 decreases in the spectra.

**Figure 4:**
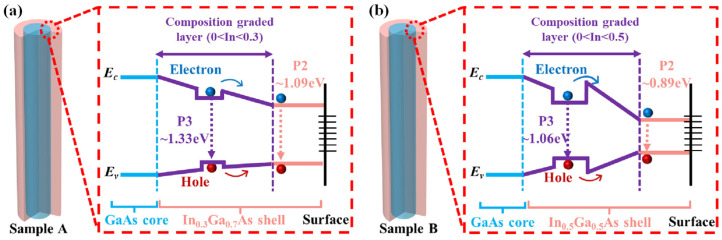
(**a**) Schematic band alignment and carrier recombination of Sample A and (**b**) Sample B.

In order to accurately verify the emission origin, the integrated intensities of P2 and P3 in two samples are extracted as shown in Figure 3c and Figure 3d, respectively. And in Figure 3c, the peak fitting was performed for two samples. The variation in integrated intensity of PL spectra with power could be represented by the following equation [38]:

(4) $$I=\;\eta I_{0}^{\alpha }$$

where *Ι_0_* is the excitation power, *η* is the emission efficiency, and the value of the power index *α* can determine the radiation mechanism. Generally, if the value of *α* is in the range of 1 < *α* < 2, the emission is attributed to the excitonic emission, while emission is generated by impurities or defects when the *α* is less than 1. By fitting the experimental data, values of the parameter α were found to be 1.04 and 1.07 for the two samples, respectively. This indicates that the P2 peak in both samples is associated with FX emission [39]. For P3 in Figure 3d, the integrated PL intensity increases almost linearly with excitation power until it approaches saturation. The excitation power saturation points are approximately 18 mW for Sample A and 25 mW for Sample B. Many reports on indium-related nanostructures have documented the presence of localized trap states [40,41,42,43]. Saturation of PL intensity, accompanied by a blue shift at high excitation power, is a common phenomenon. This behavior is typically attributed to potential fluctuations or carrier transfer in the band-tail states [44]. However, although the PL intensity of P3 tends to saturate in our system, its peak position remains fixed at approximately 1.06 eV as excitation power increases. This behavior is inconsistent with emission from localized states. Therefore, the fixed peak positions and different saturation points of P3 in two samples can be explained by the wide and deep potential well in CGL as well as by the different barrier heights in each sample, rather than by quantum well effects. The corresponding schematic band diagrams are shown in Figure 4a and Figure 4b for Samples A and B, respectively.

In the case of reverse type-I band alignment NWs, the bandgap of InGaAs shell is narrower than GaAs core (1.5192 eV, 4 K). For ternary alloy In*_x_*Ga_1−*x*_As, the bandgap of unstrained In*_x_*Ga_1−*x*_As as a function of the In composition at low temperature can be described using the empirical model proposed by Goetz et al. [45]:

(5) $$E_{g}\;=1.5192-1.5837x+0.475x^{2}$$

where *x* is the composition of In. After calculation, the bandgaps of unstrained In_0.3_Ga_0.7_As shell and In_0.5_Ga_0.5_As shell are approximately 1.077 and 0.836 eV, respectively. In our situation, these values are around 1.09 and 0.89 eV according to PL measurements of P2 in two samples. The larger bandgap values indicate that the InGaAs shell experiences compressive strain due to lattice mismatch [23]. According to previous report, the chemical bonding between In and As is much weaker than that between Ga and As; so, the movement of In atoms in the crystal matrix is much easier than those of Ga [46]. So, the CGL is probably formed by the inter-diffusion of In and Ga atoms under the growth condition of 520 °C [47]. Therefore, the In composition of CGL will be in the range of 0 < In < 0.3 and 0 < In < 0.5 for Sample A and B, respectively. The diffusion length of the atom can be denoted by the following equation according to the growth kinetics [48]:

(6) $$L=\sqrt{\tau D}$$

where *L* is the time of the atom staying in the NWs, and *D* is the diffusion coefficient of the atom. Furthermore, there is a strong dependence between the diffusion coefficient and the diffusion activation energy [41]:

(7) $$D=D_{0}exp\left(-E_{D}/kT\right)$$

where *D*_0_ is the pre-exponential factor, *E*_D_ is the diffusion activation energy, *k* is Boltzmann constant, and *T* is the temperature. In GaAs/InGaAs core–shell NWs, the rate and extent of inter-diffusion are highly dependent on the In concentration gradient [49]. In Sample B, with higher In composition, the potential well in CGL is very likely to be wider and deeper, resulting a stronger emission of P3. Furthermore, the saturation behavior in power-dependent PL may be attributed to the formation of a barrier near the potential well, caused by atomic diffusion disorder. The photon energies of P3 are approximately 1.33 eV in Sample A and 1.06 eV in Sample B. By neglecting the influence of strain, the indium composition in the potential wells of the CGL is estimated to be around 12% in Sample A and 32% in Sample B, according to Equation (2). If the In composition of the barriers is slightly lower than that of the potential wells, PL saturation is expected to occur. Additionally, the observation of saturation at higher excitation power in Sample B suggests that the barrier is higher, which means that the degree of atomic disorder is greater in Sample B than in Sample A. Therefore, the characteristics observed in the PL spectra provide evidence that P3 originates from a potential well in the CGL. This is attributed to the stronger diffusion and higher disorder of In-Ga atoms in Sample B with higher indium content.

**Figure 5:**
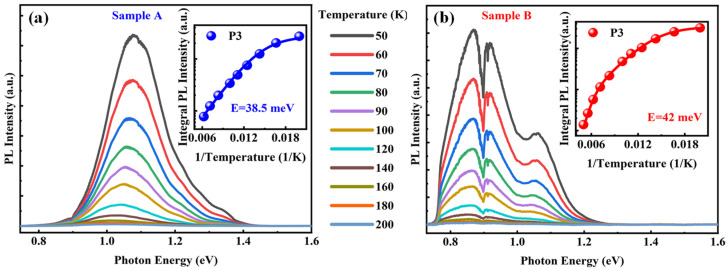
(**a**) Temperature-dependent PL spectra from 50 to 200 K of Sample A and (**b**) Sample B. The inset plots the dependence of the integrated PL intensity of P3 on the reciprocal of temperature, and the solid curve is the Arrhenius fit with one (Sample A) and two (Sample B) activation energies.

Next, we investigated the temperature dependence of PL emission from Sample A and B to gain further insight into the characteristics of potential wells and barriers. Figure 5a,b plot the temperature-dependent PL emission under an excitation power of 30 mW. As the temperature increases, a gradual decrease and redshift in PL intensity are observed. This is a typical feature of semiconductors and is likely attributed to thermal activation of nonradiative centers or the thermal escape of carriers [50]. Our analysis focuses specifically on the behavior of the P3 peak in both samples. In Sample A, P3 disappears at approximately 160 K, while in Sample B it disappears at about 200 K. The temperature-dependent integrated PL intensity can be well fitted using an Arrhenius model with a single thermal activation energy [51]:

(8) $$I\left(T\right)\;=\;\frac{I_{0}}{1+Cexp\left(-E/kT\right)}$$

where *I*_0_ is the intensity at *T* = 0 K, *E* denotes activation energy for thermal activation process, and the parameter *C* is the relative ratio of nonradiative recombination. Using a least squares fitting procedure with Equation (8), the extracted activation energies are 38.5 meV for Sample A and 42 meV for Sample B. Clarifying the physical origin of the activation process is necessary to make the fitting meaningful. The activation energy *E* can be interpreted as the energy required for carriers to be thermally excited from the potential well to the conduction band free state [52]. This means that only when the excitation energy exceeds *E* can carriers escape from the potential well in the CGL and migrate to higher energy levels. The values of *E* in two samples indicate wide energy distribution of potential well in CGL, and the slightly larger value in Sample B is correspond to the larger saturation power in power-dependent PL results. The difference in activation energies is 3.5 meV, which is consistent with the different disappearance temperatures observed in the two samples. In GaAs/InGaAs heterostructure, the conduction band (CB) offset is much larger than valence band (VB) offset, and the CB offset ratio *Q*c = (Δ*E*_c_/Δ*E*_g_) is 0.796 [53]. Based on our estimates, the In composition of the barriers adjacent to the potential wells are 9% for Sample A and 28% for Sample B. When the temperature increases to 160 K in Sample A, the electrons confined in the potential well gain sufficient thermal energy to escape from the barrier. For Sample B, this escape occurs at temperatures above 200 K. These results are consistent with those obtained from our fitting analysis.

## 4. Conclusions

In summary, we have systematically studied the optical properties of GaAs/InGaAs core–shell NWs with varying In compositions using power and temperature-dependent PL measurements. The results show that the presence of a CGL leads to the formation of potential wells whose depth and width are influenced by the In concentration profile. These potential wells significantly affect carrier confinement, PL emission features, and thermal quenching behavior. Arrhenius analysis of the temperature-dependent PL intensity indicates that the activation energies for carrier escape are consistent with the observed disappearance of specific emission peaks at elevated temperatures. These findings highlight the critical role of compositional gradient and disorder in modulating the reverse type-I energy band alignment and carrier dynamics in GaAs-InGaAs core–shell NWs, offering a valuable reference for the rational design and performance optimization of advanced nanoscale optoelectronic devices.

## Data Availability

The original contributions presented in this study are included in the article material. Further inquiries can be directed to the corresponding author.
